# Nociceptor activity is required for a robust immune response in *Drosophila* larvae

**DOI:** 10.17912/micropub.biology.002125

**Published:** 2026-06-08

**Authors:** Lydia J. Borjon, W. Daniel Tracey

**Affiliations:** 1 Department of Biology, Indiana University, Bloomington, IN, US; 2 Gill Institute for Neuroscience, Indiana University, Bloomington, IN, US

## Abstract

The nervous system and the immune system play a shared role in defending *Drosophila* larvae against injury and infection. Potentially harmful stimuli are detected by nociceptive neurons that trigger escape behaviors, while wounds and foreign invaders are sealed or killed by immune cells called lamellocytes. In this study we replicated a previous finding that silencing nociceptor output reduces the production of lamellocytes following infection by parasitoid wasps. We also investigated the timecourse of lamellocyte production to determine optional experimental time points which can be used in future investigations into the mechanism of communication between nociceptors and immune cells.

**Figure 1. Robust immune activation requires nociceptor output:** **(A)** When a wasp attacks a larva, the larva’s nociceptors activate (represented by the green neuron) and trigger rolling behavior. The attack also triggers an immune response, including the production of large numbers of lamellocytes (represented by the red cells). **(B)** Representative exemplars of MSNF9-mCherry larvae at different time points after attacks at the 2 ^nd^ instar stage. The fluorescent cells expressing MSNF9-mCherry are mature lamellocytes. The age-matched control exemplar shows that MSNF9-mCherry is also expressed in certain muscles in older larvae. Scale bars represent 1 mm. **(C)** The number of lamellocytes increases across time after larvae were attacked by wasps as 2 ^nd^ instars. n = 9-16 larvae for each time point. **(D)** The number of lamellocytes increases across time after larvae were attacked by wasps as 3 ^rd^ instars. n = 10-16 larvae for each time point. **(E)** When nociceptor output is silenced (represented by the grey neuron) during a wasp attack, the larva does not perform the rolling behavior. The immune response is impaired, resulting in the production of fewer lamellocytes (represented by the red cells). **(F)** The number of lamellocytes produced 56-66 h after attacks as 2 ^nd^ instars, in larvae whose nociceptor output is silenced by tetanus toxin (TNT-E). n = 27-46 larvae for each genotype. Chi-squared test, with Bonferroni correction, *** p>0.001, ** p>0.01, ns not significant.

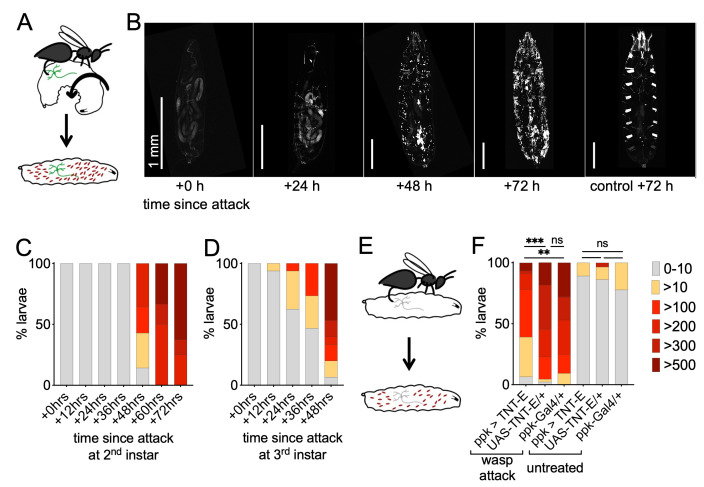



## Description

Fly larvae in the wild encounter a multitude of threats from predators, pathogens, parasites, and parasitoid wasps. The nervous system and the immune system play important roles in protecting larvae from such threats. Peripheral sensory neurons (nociceptors) that detect potentially harmful stimuli such as high temperature, harsh mechanical touch, certain chemicals and acids (Hwang et al., 2007; Lopez-Bellido et al., 2019; Tracey et al., 2003), trigger a behavioral response to help the larva escape from the source of danger ( Fig. 1A ). The escape behavior, also called rolling, consists of bending the larval body into a c-shape followed by rapid body rotations in a corkscrew-like manner (He et al., 2022; Tracey et al., 2003). Rolling locomotion may allow faster displacement away from danger than crawling, and also seems to be particularly well adapted for defending the larva against attacks by parasitoid wasps (Hwang et al., 2007; Robertson et al., 2013). The wasp’s long ovipositor, which it uses to sting the larva and lay eggs within it, becomes wrapped around the larval body like a spool, causing the wasp to be dislodged and giving the larva a chance to crawl away. However, many parasitic wasps, such as *Leptopilina boulardi* (Hymenoptera, Figitidae), have a countermeasure, a clip at the end of the ovipositor that hooks into the larval cuticle and allows the wasp to often successfully complete oviposition despite larval rolling (Buffington, 2007; Lenteren et al., 1998). In the case that a wasp egg has been deposited inside the larva, the next line of defense is the cellular immune system consisting of hemocytes that circulate in the larval hemolymph (Banerjee et al., 2019). The main cell type involved in immune defense are phagocytotic plasmatocytes that are involved in the removal of cellular debris and pathogens, and in wound healing. Crystal cells, which are a rare cell type, contribute to forming melanotic capsules at wound sites and around foreign invaders. A third type of hemocyte, lamellocytes, are also rare in healthy larvae, but greatly expand in number after an immune challenge ( Fig. 1A ). The lamellocytes that are produced in response to an immune challenge transdifferentiate from the proliferating pool of plasmatocytes in the larval periphery (Avet-Rochex et al., 2010; Honti et al., 2010; Makhijani et al., 2011; Márkus et al., 2009), or are produced in the larval lymph gland and then released into circulation (Honti et al., 2010; Lanot et al., 2001; Sorrentino et al., 2002). Mature lamellocytes are large disk-shaped cells that attach to foreign invaders, along with plasmatocytes, to form multilayered capsules which are then melanized by the crystal cells (Anderl et al., 2016; Rizki & Rizki, 1992). While the cellular immune system is canonically considered to comprise these 3 mature hemocyte subtypes, single-cell profiling now suggests that many other subtypes exist, as active cell types or as intermediate stages during maturation and transdifferentiation (Anderl et al., 2016; Cattenoz et al., 2020; Cho et al., 2020; Fu et al., 2020; Hirschhäuser et al., 2023; Tattikota et al., 2019).

Although the behavioral response and the immune response occur at different timescales, they serve a joint purpose in protecting the larva from injury and infection ( Fig. 1A ). We therefore wondered if the nociceptive system and the immune system communicate with each other. Although the majority of hemocytes circulate freely in the larval hemocoel, many plasmatocytes are also found in niches within the larval body wall (Lanot et al., 2001; Zettervall et al., 2004), at sites that are near the dendrites of sensory neurons, including near nociceptors (Makhijani et al., 2011, 2017). These sites serve as hematopoietic pockets where hemocyte proliferation and differentiation occur (Honti et al., 2010; Leitão & Sucena, 2015; Makhijani et al., 2017; Márkus et al., 2009). Hemocyte production and maturation also takes place in the larval lymph gland, but the lymph gland normally only releases those cells at the time of pupariation (Grigorian et al., 2011; Lanot et al., 2001). After wasp infection, the lymph gland disperses prematurely and also serves as a source of lamellocytes (Louradour et al., 2017; Sinenko et al., 2012), however, the major fraction of lamellocytes is derived from hematopoietic sites in the larval periphery (Makhijani et al., 2011; Márkus et al., 2009). The proximity of the hematopoietic pockets to sensory neurons may serve an important regulatory function, since the activity of sensory neurons promotes the adhesion of hemocytes to these pockets and thus influences hemocyte number (Makhijani et al., 2017). Furthermore, Tokusumi et al. showed that nociceptor activity affects the production of lamellocytes in response to wasp infections (Y. Tokusumi et al., 2017). When they blocked nociceptor output with expression of tetanus toxin, or blocked nociceptor input by mutating receptor channels, the severity of the immune response was reduced. Tokusumi et al. also reported variability in the immune response between individual larvae. In this study, we wanted to replicate the functional link between nociceptor activity and lamellocyte production, and also to build a more detailed picture of the timeline of lamellocyte production, as well as of the range of immune responses between larvae.

**Immune activation develops over several days**

First, we sought to determine how long it takes for larvae to mount a robust immune response, and to find experimental time points at which differences in lamellocyte production would be the most apparent. A commonly used protocol for wasp infections, which was also used by Tokusumi et al., incubates wasps with larvae for 24 h. This results in a high rate of infected larvae, but it could happen that some larvae escaped infection or that other larvae became superinfected with multiple wasp eggs. To avoid unknown sources of variability, we chose to closely observe the interactions between wasps and larvae, and only collected larvae where a single successful oviposition had occurred. The larvae were closely age-matched and the infections occurred within a ~1 h time window for each trial.

We then allowed the larvae to develop for different amounts of time and observed the appearance of lamellocytes by confocal microscopy ( Fig. 1B- C). To identify lamellocytes, we used the MSNF9-mCherry enhancer reporter line (T. Tokusumi et al., 2009), in which mature lamellocytes display red fluorescence. Because larvae are transparent, we were able to observe and count the lamellocytes within intact larvae immobilized between a slide and a coverslip. In addition to being marked with red fluorescence these cells are easily identified by their large disc-shaped morphology. Viewing the cells within intact larvae also made it possible to observe the cells floating freely throughout the larval hemocoel, while being spatially restricted by the internal organs such as the gut. We wondered whether it would be possible to link the location of the first lamellocytes to the location of the wasp sting, but this did not appear to be the case. Future studies could benefit from the use of automated segmentation and machine learning algorithms to detect and count lamellocytes, which would improve the throughput and accuracy of quantitative analyses. The reliability of automated methods can then be compared to manually counted datasets, such as this one.

Imaging larvae immediately after the wasp infection (+0h), all larvae were devoid of lamellocytes and contained almost no red fluorescence, and larvae continued to be almost completely devoid of fluorescence at +12h. At +24h and +36h, some red fluorescence became visible in trachea and gut, but there were still no lamellocyte-shaped cells. At +48h, lamellocytes began to appear. At this time point, there was considerable variability in the immune response of individual larvae, where some larvae still had not produced any lamellocytes, some had a few, and others already had more than 100 lamellocytes. At +60h, all the larvae had mounted a robust immune response and contained several hundred lamellocytes. By +72h, the majority of larvae had over 500 lamellocytes ( Fig. 1B- C). This time point was very close to the start of pupariation, showing that wasp-induced lamellocyte production continues all the way to the end of the larval stage. Age-matched uninfected control larvae contained very few lamellocytes, typically between 0 to 10, but occasionally more. The MSNF9-mCherry reporter also shows some expression in certain larval muscles, which is most apparent in the older control larvae ( Fig. 1B ).

**Rate of immune activation depends on larval age**

We allowed larvae to be infected at two different ages, 48h post egg lay (2 ^nd^ instar) and 72h post egg lay (early 3 ^rd^ instar). The rate at which lamellocyte number increased differed between the two age groups. While the group that was infected as 2 ^nd^ instars saw a sudden increase of lamellocytes +48h after the attacks ( Fig. 1C ), those infected as 3 ^rd^ instars began to show small but noticeable increases already after +24h ( Fig. 1D ). However, the number increased only slowly over the +36h and +48h time points. At the final time point, just over half of the larvae had mounted a robust response with several hundred lamellocytes, while some larvae still had produced very few ( Fig. 1D ). Soon after this timepoint the larvae began to pupariate. We concluded that it is optimal to perform the wasp infection on 2 ^nd^ instar larvae and to wait until around +60h in order to observe consistent robust immune activation.

**Immune activation is impaired in the absence of nociceptor output**

To replicate the finding that nociceptor activity and the cellular immune response are functionally linked, we expressed tetanus toxin (TNT) in peripheral nociceptors (class IV da neurons) (Fig.1E). Tetanus toxin cleaves synaptobrevin, thereby preventing the release of synaptic vesicles and blocking the output of the nociceptors. Silencing nociceptor signaling in this manner prevents rolling behavior in response to wasp attacks (Robertson et al., 2013). Compared to genetic controls, TNT-expressing larvae had an impaired immune response following wasp infection ( Fig. 1F ). Only about half of the larvae were able to produce more than 100 lamellocytes, and very few produced more than 300. In the genetic controls, on the other hand, the majority of larvae had mounted a robust immune response with several hundred lamellocytes, and the distribution of responses in individual larvae was significantly different compared to the experimental group ( Fig. 1F ). Uninfected controls of all genotypes contained a low baseline range of lamellocytes ( Fig. 1F ).

Therefore, our results agree with the conclusion of Tokusumi et al. (2017), nociceptor activity is required to mount a robust immune response in *Drosophila* larvae following infection by parasitoid wasps. Further, we illustrated the variability of immune responses between individual larvae, even in well-controlled conditions. The best consistency in the immune responses could be seen around +60h post wasp attack as 2 ^nd^ instar larvae, which provides a practical timeline for future investigations into the molecular mechanisms of communication between nociceptors and immune cells.

## Methods

**Fly maintenance and genotypes**

Flies were maintained on standard Bloomington Drosophila Stock Center cornmeal-agar food at 25 °C in relative humidity of 75% and on a 12h/12h light/dark cycle. The *Drosophila* strains used in this study were: *MSNF9-mCherry* (T. Tokusumi et al., 2009); *ppk1.9-Gal4* (Ainsley et al., 2003); *UAS-TNT-E* (RRID:BDSC_28837, Sweeney et al., 1995).

**Wasp maintenance**

*Leptopilina* *boulardi* strain Lb17 was maintained on Canton S *Drosophila* *melanogaster* hosts at 22 °C. Approximately 10 adult female and male flies were placed in standard cornmeal-agar food vials for 3-5 days to seed the vial with larvae. Then the adult flies were removed, and 6-8 female wasps and 2-4 male wasps were placed in the vial and allowed to infect the fly larvae for ~1 week. Any adult flies that emerged after 10-14 days were removed. Adult wasps emerged after 25-30 days and were used either for infection experiments or for the next generation of wasp cultures. Vials containing adult wasps were supplied with flugs (Genesee Scientific cat.no.49-102) dampened with honey and water (1:1 mixture).

**Infection protocol**

To collect age-matched larvae, experimental crosses of 10-20 male and female adult flies were placed into embryo collection cages. The embryo collection plates (60 x 15mm) contained 5mL of apple juice agar medium and a small amount of yeast paste (active dry yeast mixed with water). The embryo collection cages were maintained in an incubator set at 25°C and 75% humidity on a 12h/12h light/dark cycle. Embryos from restricted 4-hour egg-laying windows were allowed to develop in the incubator for 48 h until they reached the size of 2nd instar larvae, or for 72 h until they reached the size of early 3rd instar larvae.

Wasp attacks and oviposition events were observed on apple juice agar plates (60 x 15mm). 30-50 age-matched fly larvae were placed on the observation plate and allowed to acclimate for a few minutes. 12 uninfected control larvae were moved to a new apple juice agar plate. Then 6-8 female *L. boulardi* wasps were placed in the observation plate (with the lid closed) and allowed to acclimate for a few minutes. After the initial observation period, wasps that did not actively engage with the larvae were removed, leaving 2-3 wasps in the observation plate. Interactions between the wasps and larvae were watched closely through a dissecting microscope, and any larvae that were successfully attacked (the ovipositor penetrated the cuticle) were moved to a new apple juice agar plate, until at least 12 larvae were collected in a ~1 h time window. All larval transfers were performed with a flexible paintbrush and extreme caution to avoid harsh mechanical stimuli that can also trigger immune activation. The collected experimental and control larvae were allowed to continue to develop in the 25°C incubator for various durations of time, as specified in the figures.

**Microscopy and quantification of lamellocytes**

Intact larvae were imaged at each time point by placing the larva in a droplet of 100% glycerol between a glass slide and a coverslip and gently flattening the larva without causing it to burst. The larva was then imaged on an inverted confocal microscope (Zeiss LSM 5 live) at 20x magnification, illuminated by a 532 nm laser. Exemplar images were acquired as tile scans with 10x magnification. Since the lamellocytes circulated in the 3 dimensional volume of the larval hemolymph, it was easier to identify lamellocytes with live observation than from a still micrograph. Therefore, lamellocyte numbers were assessed visually through the microscope eye pieces. If fewer than 100 lamellocytes were present, the exact number was counted. If more than 100 lamellocytes were present, the volume occupied by the first 100 lamellocytes was estimated and the total number was extrapolated for the estimated remaining hemolymph volume of the larva. We estimated in ranges of more than 100, more than 200, more than 300, or more than 500 lamellocytes, acknowledging a degree of uncertainty due to an uneven distribution of lamellocytes, and challenges posed by the flow of hemolymph.

**Statistical analysis**

Data visualization and statistical analyses were performed in GraphPad Prism 10 (GraphPad Software). Comparisons between genotypes in Figure 1F were tested using the Chi-squared test with Bonferroni correction for multiple comparisons.
